# Integrated Health Care Delivery for Adolescents Living with and at Risk of HIV Infection:  A Review of Models and Actions for Implementation

**DOI:** 10.1007/s10461-022-03787-2

**Published:** 2022-07-16

**Authors:** Sujha Subramanian, Eleanor Namusoke-Magongo, Patrick Edwards, Millicent Atujuna, Teddy Chimulwa, Dorothy Dow, Emilia Jalil, Nguavese Torbunde, Kawango Agot, Ivan Arinaitwe, Jenny Beizer, Nachela Chelwa, Scovia Nalugo Mbalinda, Sam Miti, Florence Mwangwa

**Affiliations:** 1 RTI International, 3040 E. Cornwallis Road, P.O. Box 12194, Research Triangle Park, NC 27709 USA; 2 Ministry of Health, Kampala, Uganda; 3 Desmond Tutu HIV Foundation, University of Cape Town, Cape Town, South Africa; 4 Duke Global Health Institute, Durham, NC USA; 5 Fiocrus, Lapclin-AIDS, Rio de Janeiro, Brazil; 6 Institute of Human Virology, Abuja, Nigeria; 7 Impact Research and Development Organization, Kisumu, Kenya; 8 Catholic Relief Services, Kampala, Uganda; 9 Population Council, Lusaka, Zambia; 10 Makerere University, Kampala, Uganda; 11 Copperbelt University School of Medicine, Ndola, Zambia; 12 Infectious Disease Research Collaboration, Kampala, Uganda

**Keywords:** Integrated care, Adolescents, HIV care, Implementation science

## Abstract

Integrated service delivery, providing coordinated services in a convenient manner, is important in HIV prevention and treatment for adolescents as they have interconnected health care needs related to HIV care, sexual and reproductive health and disease prevention. This review aimed to (1) identify key components of adolescent-responsive integrated service delivery in low and middle-income countries, (2) describe projects that have implemented integrated models of HIV care for adolescents, and (3) develop action steps to support the implementation of sustainable integrated models. We developed an implementation science-informed conceptual framework for integrated delivery of HIV care to adolescents and applied the framework to summarize key data elements in ten studies or programs across seven countries. Key pillars of the framework included (1) the socioecological perspective, (2) community and health care system linkages, and (3) components of adolescent-focused care. The conceptual framework and action steps outlined can catalyze design, implementation, and optimization of HIV care for adolescents.

## Introduction

Adolescence is an important period of growth and development, during which the transition from childhood to adulthood involves not only physical and psychological changes but also significant life experiences like a new relationship, job, or school; a deepening sense of identity; and making decisions in a society of competing demands [1]. Although most adolescents have interconnected needs, the intersection of their health needs with the HIV epidemic requires effective interventions to reduce HIV risk, enhance early knowledge of HIV status through testing, improve adherence to treatment and offer comprehensive health services to ensure overall well-being [2–4].

Different adolescent groups have their own unique health needs [5]. Adolescent girls and young women (AGYW) living with and at risk of HIV infection not only seek HIV testing, education, and treatment services but also a range of other services for sexual and reproductive health, such as contraception, treatment for sexually transmitted infections (STIs), options to receive confidential counseling, and preventive services such as the Human Papilloma Virus (HPV) vaccination [2, 6, 7]. Besides HIV prevention and care services, young transgender men and women usually seek transgender-specific services to address medical needs related to their gender identity [8]. Adolescent boys and young men living with and at risk of HIV infection also require tailored health services, such as condom promotion and male circumcision, along with STI treatment and confidential counseling.

Studies have acknowledged the need for adolescent-responsive integrated services [9], and several projects, including the U.S. President’s Emergency Plan for AIDS Relief **(**PEPFAR) DREAMS (Determined, Resilient, Empowered, AIDS-free, Mentored and Safe) program have utilized an integrated service model [10]. The DREAMS model includes a comprehensive set of services including condom distribution, contraceptive services and care for those exposed to gender-based violence. A recent study in Zambia found that AGYW expressed a preference for a one-stop-shop for both HIV and sexual and reproductive health services. Additionally, they valued having access to care around school or other commitments, assurances of confidentiality and privacy, friendly staff who respects adolescents, and knowledgeable providers that can help adolescents to improve their health outcomes [11]. Combining gender-affirming care to HIV prevention may improve HIV prevention outcomes among transgender women, especially youth [12, 13]. By incorporating direct preferences from adolescents, integrated services can be better targeted with adolescent-focused characteristics to meet the complex needs of adolescents [11]. Although many of these health services are offered directly to adolescents in the clinic setting, offering support in the community setting and reinforcing beneficial strategies through public policy may help to enhance linkages to care and optimize programs for improved HIV outcomes [7]. Furthermore, implementation science based frameworks and methods should be further reinforced in adolescent studies to successfully embed evidence-based interventions and strategies into the practice setting.

Although adolescent-responsive integrated service delivery approaches have been advocated, there is no comprehensive assessment of past and ongoing projects to guide the adoption of optimal models. The purpose of this study is to offer guidance for future program design, implementation and evaluation of adolescent responsive integrated programs. First, we present an implementation science-informed conceptual framework to support the development of comprehensive adolescent-responsive integrated service delivery models in HIV care. Secondly, we summarized characteristics of selected projects that have implemented integrated delivery of health services targeted at adolescents living with and at risk of HIV infection to catalogue features of existing integrated models to identify implementation gaps. Third, we develop action steps to support the implementation and evaluation of adolescent-responsive integrated programs drawing on implementation science theoretical approaches focusing on implementation outcomes and determinants.

## Methods

### Develop Conceptual Framework

We used a three-step process to develop a tailored conceptual framework focused specifically on the integrated delivery of care to adolescents within HIV care programs. First, we identified key themes on barriers and preferences related to health care service delivery from prior work done by this team to inform this conceptual framework [11]. We had previously conducted interviews and focus groups with 109 AGYW aged 10 to 24 years in Zambia as part of formative research conducted for planning an implementation science trial [7]. The participants included both AGYW living with HIV and those at risk of HIV infection. Furthermore, we undertook two rounds of consultation with the six-member youth advisory board that consisted of both young men and women and was initiated for the above-mentioned study. We developed detailed guides and conducted systematic qualitative analysis to identify emergent themes. These details are included in a recently published manuscript [11]. Second, we created an initial framework that was presented to implementation scientists and researchers attending the Joint Adolescent HIV Prevention and Treatment Implementation Science Alliance (AHISA) and Prevention and Treatment through a Comprehensive Care Continuum for HIV-affected Adolescents in Resource Constrained Settings (PATC^3^H) meeting in February 2020. The AHISA consortium and the PATC^3^H program include teams of researchers and implementers working to advance implementation science-based research focused on adolescents and young adults living with or at risk of HIV infection. Feedback received through interactive discussion was incorporated to create an updated framework. Third, we convened a virtual forum via Zoom with individuals implementing health care service delivery programs for adolescents living with and at risk of HIV infection (including the manuscript team representing Brazil, Kenya, Nigeria, South Africa, Tanzania, Uganda and Zambia) to clarify key themes and further refine the framework components. Through this forum, we also adapted guidance published by the World Health Organization (WHO) to highlight components of adolescent-responsive care that should be included to implement these programs successfully.

### Review Selected Projects

We initiated a review of programs to identify examples of adolescent-targeted projects to summarize models currently being implemented. Our inclusion criteria were projects that targeted adolescents at risk of or living with HIV for HIV testing or HIV treatment, respectively, and integrated at least one non-HIV-related service. Health care services could include reproductive health, new-born care, gender-affirming care, mental health, substance use services, services for gender-based violence and violence against children, and preventive care (including screenings and vaccinations). We asked study co-authors to submit research projects from their respective countries. Using the conceptual framework, we identified key data elements and measures to compare the selected projects. We report on the populations targeted, socio-ecological levels addressed, and services provided. Given the importance of program setting for implementation science evaluations, we also provide details on contextual factors including HIV prevalence, income level and guidelines related to services for adolescents living with and at risk of HIV infection.

### Create Action Steps

In further discussion during the virtual forum described above, participants identified action steps and components for planning, implementing and evaluating integrated adolescent-responsive programs. These were summarized in an “Action Plan” to support adoption of integrated adolescent services.

## Framework for Developing and Implementing Adolescent-Focused Integrated Modular Services

**Fig. 1:**
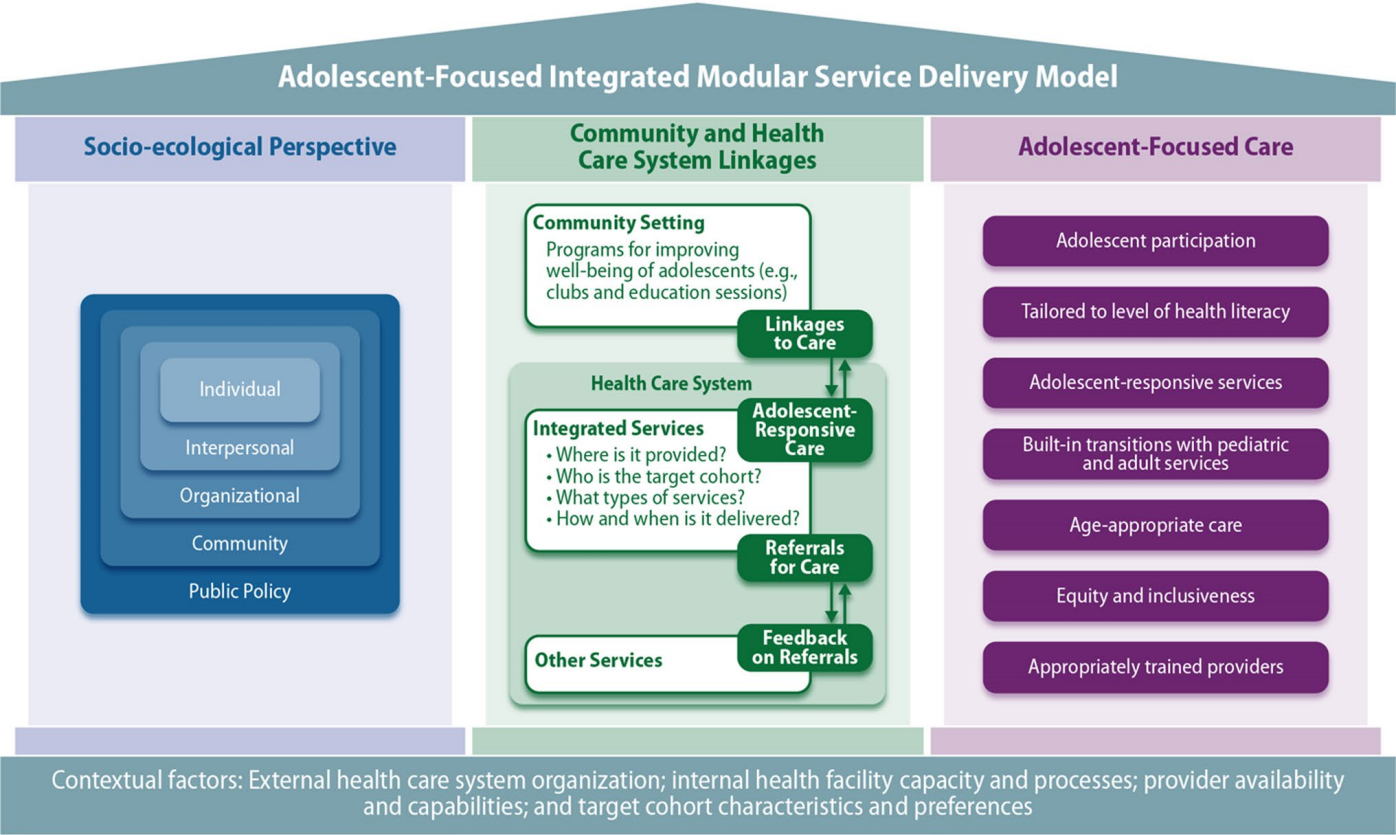
Framework for developing and implementing adolescent-focused integrated modular services (AIMS)

The major thematic areas identified from the feedback with adolescents and young adults and consultation with HIV program implementers included: (1) the importance of incorporating multilevel barriers that influence adolescent care seeking behavior and the use of services; (2) community-clinic linkages that include education and other community engagement; (3) integration of services and referral pathways for specialty care; and (4) the importance of ensuring services that are tailored to adolescents. Figure 1 presents the framework that we created for developing and optimizing Adolescent-focused Integrated Modular Services (AIMS) that includes three key pillars based on thematic areas identified. The first pillar, “the socio-ecological perspective,” demonstrates that adolescents operate in an interrelated network that includes many stakeholders who can positively and negatively influence adolescent well-being and that barriers to optimal care exist at multiple levels. Interventions and implementation strategies based on the socio-ecological perspectives can be targeted at the individual, interpersonal, organizational, community, and public policy levels. As barriers faced by adolescents can be experienced at each of these levels, successful programs will need to implement multilevel strategies to address these barriers. The second pillar, “community and health care system linkages,” includes processes and features in the community and health care setting and linkages and feedback mechanisms. Programs within the community setting, such as youth clubs, can be leveraged to encourage linkages to integrated adolescent-responsive services within the health care system and can provide referrals to other services or supports within the health system. Integrated services can be delivered using a modular approach that will allow services to be tailored based on ‘who’, which cohort of adolescents, is being targeted. This can guide ‘what’ services are offered, ‘where’ the services are provided, and ‘how’ and ‘when’ the services are offered. The third pillar, “adolescent-focused care,” includes seven tailored elements of care that ensure interventions and services appropriately meet the unique needs of adolescents. These adolescent-focused care elements include adolescent participation, services tailored to adolescents’ level of health literacy, adolescent-responsive services, built-in transitions with pediatric and adult services, age-appropriate care, equity and inclusiveness, and appropriately trained providers. The definitions of key concepts from the framework are included in Table 1. The framework also includes contextual factors, which are features external to the adolescent-responsive program that may shape service delivery and sustainability. Altogether, the components of the AIMS framework relate to each other by including both internal and external attributes across multiple levels of society that can influence the implementation and uptake of adolescent-responsive integrated services. The AIMS framework provides a structured approach to design and develop integrated programs for adolescents that can be tested using implementation science-based trials.

**Table 1:** Definitions of concepts in AIMS framework

Construct	Definition and description
Socio-ecological perspective	
Individual	Knowledge, attitude, self-efficacy
Interpersonal	Peer group, friends, family, support groups
Organizational	Health system facilities, schools, youth associations
Community	Safe spaces, cultural attitudes, transportation
Public Policy	Guidelines, laws, and procedures at the national, regional, and local levels
Integrated care in the community and health care system	
Where is it provided?	Whether service is offered in the community or health care setting and by facility type (e.g., public health centers, private clinics, pharmacies, mobile clinics, youth centers and schools)
Who is the target cohort?	Specific cohorts could include specific gender (e.g., girls only) or special populations (e.g., HIV-positive adolescents only)
What type of services?	Could include a range of services, including sexual reproductive health, HIV testing, HIV treatment, and mental health or substance abuse counseling
How and when is it delivered?	Details on who is delivered the required services, timing of clinic opening hours, clinic location, use of eHealth or mHealth
Adolescent-focused care	
Adolescent participation	Adolescents are involved in planning, monitoring and evaluating health services and in decisions regarding their own care, as well as in certain appropriate aspects of service provision
Tailored to level of health literacy	The health facility implements systems to ensure that adolescents are knowledgeable about their own health and they know where and when to obtain health services
Adolescent-responsive services	The health facility has convenient operating hours, provides a welcoming and clean environment and maintains privacy and confidentiality. It has the equipment, medicines, supplies and technology needed to ensure effective service provision to adolescents
Built-in transitions with pediatric and adult services	The health facility implements systems to ensure adolescents can access the appropriate package of pediatric services, including through referral linkages and outreach along with planning, coordinating, and transitioning to adult services
Age-appropriate care	Adolescence is a time of transition and individual needs should be considered through this transition; ethical, legal, and cultural factors will have to be considered to ensure appropriate care is provided. For example, at what age issues related to sexual and reproductive health are appropriate
Equity and inclusiveness	The health facility provides quality services to all adolescents irrespective of their ability to pay, age, gender, marital status, education level, ethnic origin, sexual orientation, or other characteristics
Appropriately trained providers	Health care providers demonstrate the technical competence required to provide effective health services to adolescents. Both health care providers and support staff respect, protect, and fulfil adolescents’ rights to information, privacy, confidentiality, non-discrimination, non-judgmental attitude, and respect

*AIMS* adolescent-focused integrated modular services

## Summary of Selected Programs Using Aims Framework

We identified adolescent HIV programs with integrated service delivery approaches in seven countries. Selected contextual factors of the countries where the programs are implemented are presented in Table 2. Six out of the seven countries included are located in Sub-Saharan Africa (Zambia, Kenya, Nigeria, Uganda, Tanzania, and South Africa) while the remaining country (Brazil) is located in South America. We provide the income classification, along with the income level, for each country as this may impact the resources available for adolescent-responsive programs [14, 15]. Uganda is classified as a low-income country; Zambia, Kenya, Nigeria, and Tanzania are classified as lower-middle income countries; and Brazil and South Africa are classified as upper-middle income countries. The adolescent (ages 15–24) HIV prevalence across the included countries (excluding Brazil as no estimates were available) ranges from a low of 0.4% (Nigeria) to a high of 6.9% (South Africa), and the adolescent HIV incidence (per 1000 aged 15–24) ranges from 0.57 (Nigeria) to 8.97 (South Africa) [16]. Among Brazilian young transgender women, HIV prevalence was 24.5% [5]. Updated adolescent HIV guidelines were all developed within the last 5 years for each of the countries [17–23]. Both Nigeria and Uganda’s guidelines included various elements from the conceptual framework, including adolescent-responsive services, the socio-ecological perspective, gender, community engagement, and health literacy. The most recently available general health guidelines for adolescents were largely developed between 2012 and 2018 for each country, with three countries (Kenya, Nigeria, and Uganda) explicitly including elements of adolescent-focused care from the framework [24–30]. Adolescent multi-sectoral guidelines were also found for Kenya, Brazil, South Africa and Tanzania and included HIV education in schools and HIV guidelines for key and vulnerable populations [31–36].

**Table 2:** Setting of countries included in review (contextual factors in the AIMS framework)

Country	Programs that link to or provide adolescent care	Geographic location	Income category [14] [per capita income—USD] [15]	Adolescent HIV prevalence (15–24 years) [16] [range]	Adolescent HIV incidence per 1000 (15–24 years) [16] [range]	National guidelines established (Latest year for each)		
						Adolescent HIV guidelines	Adolescent general health guidelines	Adolescent multi-sectoral guidelines
Zambia	DREAMS, SHIELD/IWC	Sub-Saharan Africa	Lower–middle [$1305]	4.2 [2.5–5.6]	6.35 [4.09–8.53]	2020 [17]	2015 [24]	*–*
Kenya	DREAMS	Sub-Saharan Africa	Lower–middle [$1817]	1.7 [1.2–2.2]	1.25 [0.73–2.15]	2018 [18]	2014 [25] (Adolescent-responsive services, community engagement, transition with pediatric and adult services)	2016 [31] (Adolescent-responsive services, community engagement)
Nigeria	OTZ, Zvandiri Program	Sub-Saharan Africa	Lower–middle [$2230]	0.4 [0.2–0.7]	0.57 [0.27–1.14]	2020 [19] (Adolescent-responsive services, socio-ecological perspective, integrated services, gender)	2018 [26] (Adolescent participation, community engagement)	
Uganda	DREAMS, YAPS, G-ANC	Sub-Saharan Africa	Low [$2220]	1.9 [1.2–2.5]	1.63 [1.06–2.24]	2020 [20] (Community engagement, health literacy)	2012 [27] (Adolescent-responsive services)	
Brazil	BeT	South America	Upper–middle [$8717]	Overall estimates not available 24.5% (among young transgender women) [5]	No estimates are available	2018 [21]	2018 [28]	2010 [32] 2013(HIV specific) [33]
Tanzania	DREAMS, SYV	Sub-Saharan Africa	Lower–middle [$1,122]	1.7 [1.0–2.3]	1.86 [1.16–2.42]	2019 [22] National HIV guidelines	2018 [29]	2002 [34] 2017 [35]
South Africa	Beyond Zero, IMARA, DREAMS	Sub-Saharan Africa	Upper–middle [$6,748]	6.9 [3.0–10.7]	8.97 [4.08–14.17]	2020 [23]	2017 [30]	2020 [36]

*AIMS* adolescent-focused integrated modular services; *DREAMS* Determined, Resilient, Empowered, AIDS-free, mentored and safe; *SHIELD-IWC* support for HIV integrated education, linkages to care, and destigmatization/integrated wellness care; *OTZ* operation triple zero; *YAPS* young people and adolescent peer support; *G-ANC* Group Antenatal Care/Postnatal Care; *BeT* “Brillar e Transcender” (English: “Shine and Transcend”); *SYV* Sauti Ya Vijana; *IMARA* informed motivated aware responsible adolescents and adults

Across the seven countries, we identified 10 adolescent programs which included DREAMS; Support for HIV Integrated Education, Linkages to care, and Destigmatization/Integrated Wellness Care (SHIELD/IWC); Operation Triple Zero (OTZ); Zvandiri Program; Young People and Adolescent Peer Support (YAPS); Group Antenatal Care/Postnatal Care (G-ANC); “Brillar e Transcender” (BeT; English: “Shine and Transcend”); Sauti Ya Vijana (SYV); Beyond Zero; and IMARA (Informed Motivated Aware Responsible Adolescents and Adults) [7, 10, 37–44]. The socio-ecological perspective, first pillar in the AIMS framework, is presented in Table 3 for the programs identified.. Five of the selected programs focused specifically on AGYW (DREAMS, SHIELD/IWC, G-ANC, Beyond Zero, and IMARA), while one program focused on young transgender women ages 18 to 24 (BeT). The other four programs included both adolescent boys and girls largely between the ages of 10 to 24 years (OTZ, Zvandiri Program, YAPS, and SYV). Across the interpersonal level of the socio-ecological perspective, nine of the ten programs included caregivers, and many also included peers, support groups, and partners. Organizationally, seven programs were largely at the health clinic/provider level, one was based out of schools, and another out of the Ministries of Health, Education, and Sports program. To foster linkages to the services offered, most programs included various community mobilization programs, peer navigators, campaigns, digital outreach, and other support networks. At the public policy level, most projects were influenced by or operated in accordance with a national policy such as Ministry of Health and other national guidelines.

**Table 3:** Socio-ecological perspectives of selected adolescent programs (first pillar in AIMS framework)

Program name	Socio-ecological levels				
	Individual	Interpersonal	Organizational	Community	Public policy
DREAMS	AGYW ages 10–24	AGYW’s partners, Caregivers/family	School-based programs	Community mobilization and norms change programs	Public/private partnerships across member countries
SHIELD/IWC	AGYW ages 10–25	Caregivers/family	Health clinics/clinic providers	Peer navigators/community support	Ministry of Health and national guidelines
OTZ	Adolescents and young people (AYP) ages 10–24	Peers living with HIV, caregivers, AYP support groups	Health clinics/clinic providers	Enrollment in the Orphans and Vulnerable Children’s program	National HIV and Adolescent and Youth Friendly Health Services (AYFHS) guidelines
Zvandiri Program	Adolescents ages 10–19 years	Peers, caregivers, family, support groups	Health clinic/clinic providers	Linkages between community and facilities, Radio program engaging adolescents, HIV self-testing in the community	National HIV and AYFHS guidelines
YAPS	Adolescents and young people (girls and boys) ages 10–24 years	Care givers, peers, family members, sexual partners	Ministries of Health, Gender, Labour and Social Development, and education and sports	Village health teams, local council, para-social workers, paralegals, community-based organizations, faith-based organizations, religious institutions, schools, people living with HIV networks	National Adolescent Health Policy and service standards; Consolidated guidelines for prevention and treatment of HIV; National Guidelines for Differentiated service delivery
G-ANC	Pregnant and lactating AGYW ages 15–24 years	Care givers, peers, family members, sexual partners	Health care workers at health facilities	AGYW safe spaces in the community, community-based organizations, faith-based organizations, paralegals	National Reproductive, Maternal, Newborn, Child and Adolescent Health (RMNCAH) Guidelines
BeT	Young transgender women ages 18–24	None	Health clinics/clinic providers	Anti-stigma campaign/Peer health digital navigation	Ministry of Health and National PrEP implementation project
SYV Tanzania	10–24 years with a focus on 15–24 years (AYA living with HIV)	Caregivers/supportive adults	Adolescent HIV clinics	Peer group leaders and youth community advisory board	Engagement with MOH with policy brief to influence importance of adolescent mental health in HIV prevention and treatment programming
Beyond Zero	AGYW 15–24 years old	Teens and caregivers	Health clinics	Return to school, economic strengthening, opportunities for bursaries, CSE institutional support, academic support	Operates in 3 provinces in South Africa with local partners on the ground in these communities
IMARA	AGYW 15–19 years old	Family based, with a focus on Female care givers,	Family based programs, Lay facilitators delivering the intervention	Gender dynamics, partner relationships, HIV stigma and discrimination	Engagement with Provincial department of health, social development

*AIMS* Adolescent-Focused Integrated Modular Services; *DREAMS* Determined, Resilient, Empowered, AIDS-free, Mentored and Safe; *SHIELD-IWC* Support for HIV Integrated Education, Linkages to care, and Destigmatization/Integrated Wellness Care; *OTZ* Operation Triple Zero; *YAPS* Young People and Adolescent Peer Support; *G-ANC* Group Antenatal Care/Postnatal Care; *BeT* “Brillar e Transcender” (English: “Shine and Transcend”); *SYV* Sauti Ya Vijana; IMARA Informed Motivated Aware Responsible Adolescents and Adults; *AGYW* Adolescents Girls and Young Women; *AYP* Adolescents and Young People; *AYFHS* Adolescent and Youth Friendly Health Services

Health services provided along with the community-health system linkages, thesecond pillar in the AIMS framework, are listed in Table 4. Each program offered a range of health services tailored toward the needs of the adolescents included in each program. These services generally included HIV prevention and treatment services (e.g., HIV testing and counseling, pre-exposure prophylaxis [PrEP], and adherence counseling), and some also included family planning services (e.g., condom promotion, contraceptives, pregnancy testing) and adolescent-specific days or hours. Additionally, antenatal and postnatal services (one program), mental health (one program), HPV vaccination (one program), or other supportive services like education subsidies or literacy trainings were also provided. Eight programs also offered community-health system linkages, including peer navigation, youth clubs, school programs, non-governmental organization referrals, or caregiver programs.

**Table 4:** Community and healthcare system services and linkages of selected adolescent programs (second pillar in AIMS framework)

Program name	HIV Testing/counseling	Anti-retroviral adherence counseling	Disclosure counseling	PrEP	Condom promotion	Pregnancy testing/SRH services	Peer navigation/support/mentorship	Educational programs	Other activities
DREAMS	X			X	X			X (adolescents and caregivers)	Community/social media engagement, violence prevention and care
SHIELD/IWC	X	X			X	X	X	X (adolescents and caregivers)	HPV vaccination, contraceptives, referrals
OTZ		X	X				X	X (adolescents and caregivers)	Age-band services, community/social media engagement, psychosocial support, viremia clinics
Zvandiri Program		X	X				X		Age-band services, community/social media engagement
YAPS Program	X	X	X	X			X	X (adolescents)	Age-band services, community/social media engagement, appointment monitoring, tracking and follow-up, school linkages
G-ANC						X (ANC/PNC care)			
BeT	X	X		X			X		Gender-affirming hormones, mental and endocrinological care
SYV Tanzania		X			X			X (adolescents)	Mental health and life skills, referrals
Beyond Zero	X	X	X	X	X	X	X	X	In school and out of school programs demand creation, and biomedical services
IMARA	X		X	X	X			X (family-based)	ART referrals, partner notification, STI testing and treatment, mental health education

*AIMS* Adolescent-Focused Integrated Modular Services; *PrEP* pre-exposure prophylaxis; *SRH* Sexual and Reproductive Services; *DREAMS* Determined, Resilient, Empowered, AIDS-free, Mentored and Safe; *SHIELD-IWC* Support for HIV Integrated Education, Linkages to care, and Destigmatization/Integrated Wellness Care; *OTZ* Operation Triple Zero; YAPS- Young People and Adolescent Peer Support; *G-ANC* Group Antenatal Care/Postnatal Care; *BeT* “Brillar e Transcender” (English: “Shine and Transcend”); *SYV* Sauti Ya Vijana; *IMARA* Informed Motivated Aware Responsible Adolescents and Adults; *HPV* Human Papillomavirus; *ART* antiretroviral treatment; *STI* sexually transmitted infection

Adolescent-focused care elements, the third pillar of the AIMS framework, are presented in Table 5 for each of the included programs. All programs had some degree of adolescent participation through either a delivery or advisory role. Nine of the programs specifically tailored their content to the health literacy of the adolescents included. Eight programs specifically tailored their intervention to be adolescent-responsive by providing services with convenient opening hours, dedicating time and space for adolescents while maintaining privacy and confidentiality, and providing integrated services tailored towards the need of adolescents. Five programs offered built-in transitions with pediatric and adult services, all ten explicitly ensured age-appropriate care, seven programs included a focus on equity and inclusiveness (by age, gender, HIV status), and all ten ensured having appropriately trained providers.

**Table 5:** Elements of adolescent-focused care among selected programs (third pillar in AIMS framework)

Program name	Adolescent-focused care elements						
	Adolescent participation	Tailored to level of health literacy	Adolescent-responsive services	Built-in transitions with pediatric and adult services	Age-appropriate care	Equity and inclusiveness	Appropriately trained providers
DREAMS	X (delivery)	X	X (with referrals to facilities)		X	X	X (mentors receive training)
SHIELD/IWC	X (advisory)	X	X (integrated)		X	X (age, gender, HIV status)	X (stigma, adolescent-responsive care)
OTZ	X (delivery)	X	X (dedicated time & space)	X	X		X
Zvandiri Program	X	X	X	X	X		X
YAPS Program	X (delivery)	X		X	X	X	X
G-ANC	X		X	X	X	X	X
BeT	X	X	X		X	X (gender)	X
SYV Tanzania	X (delivery)	X		X	X		X
Beyond Zero	X (advisory)	X	X		X	X (have other programs for men who have sex with men and transgender adults, 15–49 years	X (clinic staff and counsellors)
IMARA	X	X	X		X	X (HIV status, sexually active and non-sexually active girls)	X (Nurse, adolescent trained counsellors, facilitators)

*AIMS* Adolescent-Focused Integrated Modular Services; *DREAMS* Determined, Resilient, Empowered, AIDS-free, Mentored and Safe; *SHIELD-IWC* Support for HIV Integrated Education, Linkages to care, and Destigmatization/Integrated Wellness Care; *OTZ* Operation Triple Zero; *YAPS* Young People and Adolescent Peer Support; *G-ANC* Group Antenatal Care/Postnatal Care; *BeT* “Brillar e Transcender” (English: “Shine and Transcend”); *SYV* Sauti Ya Vijana; *IMARA* Informed Motivated Aware Responsible Adolescents and Adults

## Action Plan for Implementing and Evaluating Aims Programs

**Fig. 2:**
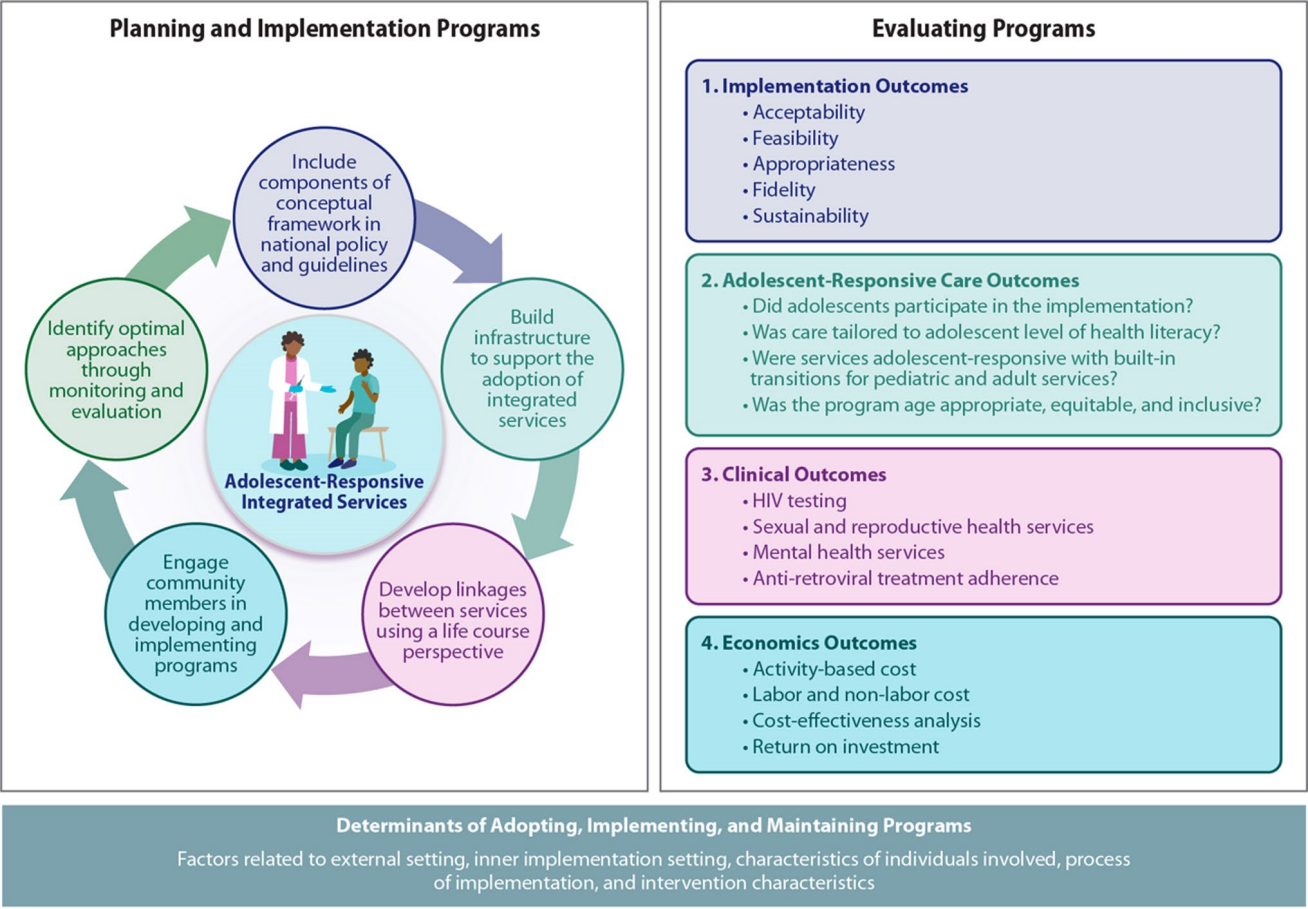
Action plan to support adoption of adolescent-responsive integrated services for HIV-affected populations using an implementation science approach

Key actions to support the adoption of adolescent-responsive integrated services for populations living with and at risk of HIV infection are presented in Fig. 2. The actions consist of (1) including components of the AIMS conceptual framework in national policy and guidelines; (2) building infrastructure to support the adoption of integrated services; (3) developing linkages between services using a life course perspective; (4) engaging community members in developing and implementing programs; and (5) identifying optimal approaches through program monitoring and evaluation. These actions are essential components to ensure clear, policy-oriented steps are in place to support the processes required to implement, assess, and modify approaches to ensure sustainable program models that deliver optimized services to adolescents.

The first step is to ensure the inclusion of key components of the AIMS conceptual framework for integrated adolescent-responsive services in national policy and guidelines. National policy should acknowledge the multiple levels of influence on adolescent health, adopt a process to foster community-clinic linkages, identify services that will be integrated, develop referral pathways for other specialty services, and support all elements of adolescent-focused care such as adolescent participation and education materials tailored to age-appropriate health literacy levels.

Second, it is essential to build infrastructure to support the adoption of integrated services. HIV care services for adolescents should be strengthened along six key indicators, identified by WHO [45], which make for strong health-systems, including service delivery, governance, health workforce, information systems, medical products (e.g., medicines, vaccines, and technology), and financing.

The third action step is to develop linkages between services using a life course perspective. Adolescent care requires intentional planning to ensure smooth transitions from pediatric care to adult care. These transitions should focus beyond HIV services to allow for transitions in integrated services such as sexual and reproductive health.

Fourth, community members and key stakeholders should be engaged in developing and implementing programs. Adolescents, their caregivers, and community leaders can offer valuable insights to generate demand for services offered, and providers involved in care processes can help design appropriate services to ensure adolescent-responsive supply-side procedures.

Fifth, a critical component is ensuring a continual learning system by identifying optimal approaches through monitoring and evaluating the implementation processes and outcomes.

Figure 2 also provides four components that should be included in comprehensive implementation science-based evaluations comprising of implementation outcomes, adolescent-focused care outcomes, relevant clinical outcomes and economics outcomes. The evaluation components specifically include adolescent responsive measures to assess implementation of adolescent-focused care identified in Fig. 1. Furthermore, the action plan highlights the importance of considering external and internal determinants of successfully adapting, implementing and maintaining programs.

## Discussion

We have presented a comprehensive framework to guide the design and implementation of adolescent-responsive integrated services. The AIMS framework includes three pillars: the socioecological perspective to highlight the importance of multilevel strategies; modular set of integrated services in the community and clinic settings that can be tailored to the target cohort and implementation setting; and comprehensive set of practices to deliver adolescent-focused care. Adolescents living with and at risk of HIV infection need services beyond those directly related to HIV [46]. Support services such as those related to sexual health and reproductive services and mental health can supplement efforts to prevent and treat HIV among adolescents. Fortunately, there is growing recognition of the need for integration and there are many ongoing programs and initiatives, including those reviewed in this study. Examples of additional programs include Ariel adherence clubs, REACH (Re-Engage Adolescents and Children with HIV) and Baylor College of Medicine International Pediatric AIDS Initiative Teen Club Programme [47, 48]. The AIMS framework offers standardized components and definitions to compare and evaluate programs delivering services to adolescents living with or at risk of HIV infection to optimize integrated care delivery.

Several of the guidelines reviewed for this study include components of adolescent-responsive care but additional advocacy is required to ensure the comprehensive inclusion of components across all guidelines. Importantly, guidelines do not specifically address integrated delivery of services for adolescents and action steps based on implementation science approaches outlined in this manuscript can support the implementation and evaluation of integrated service delivery programs. Furthermore, COVID-19 has disrupted health care access and reduced number of in-person visits which has highlighted the importance of offering integrated one-stop-shop services to maximize the reach of care essential for adolescent wellbeing [49]. The AIMS framework offers structured components to support the inclusion of adolescent-responsive integrated care models in guidelines for ameliorating COVID-19 pandemic impacts on adolescents living with or at risk of HIV infection. As indicated in the AIMS framework, adolescent participation is essential in the design, implementation and evaluation of these programs [50].

This review of selected projects focused on implementing integrated services for adolescents at risk of and living with HIV and identified several gaps that should be addressed in future programs and studies. First, research is needed to identify the appropriate mix of services for the targeted population in terms of what services should be offered in a single visit or in one setting and which specialty services should be provided via referrals. Adolescent girls and adolescent boys each require a different service mix whereas other groups such as transgender adolescents as well as pregnant adolescents may need additional types of care. Second, evidence-based multilevel strategies are needed to support adolescent-responsive services. For example, many of the projects reviewed in this study are implementing peer navigation and education support but these strategies may not address barriers faced at multiple levels of the socioecological model. Systematic implementation science research is required to identify which strategies and features of each strategy work best for targeted adolescent cohorts. Additionally, as outlined in Fig. 2, standardized implementation outcomes such as those related to acceptability and appropriateness should be collected across projects to optimize the delivery of adolescent-responsive components. Third, to support comparison across projects offering integrated services, we need outcome measures beyond the assessment of impact along the HIV continuum. Outcome measures and metrics that currently exist for HIV [51] should be expanded to capture services that impact other important domains such as sexual and reproductive health and mental health. Fourth, cost-effectiveness assessments should be incorporated as a key research component in all projects offering integrated services to adolescents so policy makers can identify the most efficient use of available resources.

## Conclusion

Despite resource constraints and capacity challenges, countries with populations disproportionately impacted by HIV are attempting to implement programs to improve delivery of adolescent health care services. By adopting a life course perspective and recognizing that adolescent-focused care is a vital link between pediatric and adult health services, governments can adopt tailored strategies to ensure adolescents use available services with support from their caregivers. The conceptual framework and action steps outlined in this manuscript can serve as an important catalyst to design, implement, and optimize adolescent-responsive services for those living with and at risk of HIV infection.
